# A Manual of Medical Jurisprudence, &c.

**Published:** 1831

**Authors:** 


A Manual of Medical Jurisprudence, &c. By Michael Ryan, M.D. Octavo, pp. 309. 1831.

This work, which is an elementary compilation from the best authors, interspersed with many original and acute observations, we can only notice briefly, since it is as incapable of being analyzed as a dictionary. If the author only laid claim to the merit of industry, that merit would be not a little laudable; but he is, in fact, possessed of great talent as a learned writer, a judicious compiler, and an instructive lecturer. The ability and diligence with which he conducts our contemporary, the Medical and Surgical Journal, have made him quickly and very favourably known throughout the profession—and the versatility of his talents as an author and teacher, is exceedingly creditable in so young a man.

Dr. Ryan's object, in this cheap and well-constructed volume, is to give a concise, yet comprehensive view of the received principles of medical jurisprudence, and to collect, in a small compass, the scattered and isolated facts from the standard works of legal and medical writers. In many portions of the work, however, Dr. R. is not a mere compiler; for, in both the ethical and legal chapters, he has interwoven a great deal of original—and, what is of more consequence, of enlightened, liberal, and independent remarks, which cannot fail, if duly appreciated, to be of great use to all classes of the profession, but more especially to the junior members. The laws relating to the different orders of the faculty, in these kingdoms, are more clearly enunciated, and more succinctly compiled, than in any other publication in the English or any other language. In the sections on medical evidence and the adulteration of alimentary matters, much original and important information is concentrated in a small space.

"In exposing (says he) the absurd distinctions, the defective state of the laws relating to the profession, and the gross abuses of its constituted authorities, the love of freedom and of equality, with an ardent desire to promote the interests of his favourite science and of humanity, have impelled him to declare the truth, however unpalatable that may be in certain quarters, or to the different orders of the faculty. His motto has been, 'amicus Socrates, amicus Plato, sed magis amica veritas.' He has not been the advocate of any party, of any order, of any corporation, but the advocate of the whole profession."

We have, indeed, been astonished that Dr. Ryan has been able to dedicate so much time to the laborious research which this volume displays, considering his other avocations as an editor, a lecturer, and a practitioner.

We will not maintain that the work is without imperfection—for what work could fairly claim such distinction? Nor do we entirely agree with Dr. Ryan in all his ethical precepts. The author, indeed, from want of attention to inverted commas, has sometimes enunciated opinions which do not breathe the liberal spirit with which he himself is inspired. The following passage, though not marked as a quotation, is, we are convinced, not the sentiment of the author.

"The presence of an apothecary at a consultation can be of no use whatever to the patient, and is very often injurious. Physicians, in his presence, cannot deliberate as freely as they would do, were they by themselves. They feel that they are under the *surveillance* of a person who may have a partiality towards one physician, and a prejudice against another, and who may pass what comment he pleases on their opinions and practice. The effect of this is, to create a degree of caution and reserve on their part, altogether inconsistent with the object of a consultation; and which often renders it little else than a mere matter of form!"*

If the apothecary of our own time was the mere compounder of drugs, as he was thirty or forty years ago, and uninitiated in the principles and practice of therapeutics, there might be some ground for the foregoing observation. But the general practitioner (for there is now no such thing as mere apothecary in this country) is well educated, and imbued with great practical knowledge, from daily observation of diseases in all their varied forms—he is, therefore a very desirable, and often an indispensible personage in the consultation, where the good of the patient, rather than the academic pride of the physician, is to be taken into account. Our intercourse with this class is probably as great as that of the generality of our contemporaries, and we can conscientiously declare that we would be very sorry, in any important case, to be deprived of the assistance and advantage of the attendant practitioner.

We have made these observations, and we have quoted the above passage to shew that we do not praise Dr. Ryan's work as a mere compliment, and without having perused it. But, we may add, the errors and blemishes are very few indeed, and scarcely sully the peformance, which, as a whole, does great credit to the author's heart, as well as his head. After this declaration, it is hardly necessary to say, that we recommend the work as being, all that it professes to be, a MANUAL OF MEDICAL JURISPRUDENCE.

P.S. We shall revert to particular parts of the work in our next Number.

---

* We believe that this passage is attributable to Dr. Grattan, of Dublin; and it is a pity that Dr. Ryan did not consign the property to its real owner, since it is not worthy of being either borrowed or stolen.

